# ORAI1 Ca^2+^ Channel as a Therapeutic Target in Pathological Vascular Remodelling

**DOI:** 10.3389/fcell.2021.653812

**Published:** 2021-04-06

**Authors:** Heba Shawer, Katherine Norman, Chew W. Cheng, Richard Foster, David J. Beech, Marc A. Bailey

**Affiliations:** ^1^School of Medicine, The Leeds Institute of Cardiovascular and Metabolic Medicine, University of Leeds, Leeds, United Kingdom; ^2^School of Chemistry, University of Leeds, Leeds, United Kingdom

**Keywords:** ORAI1, STIM1, calcium, vascular remodelling, store operated calcium entry, vascular smooth muscle, pharmacology

## Abstract

In the adult, vascular smooth muscle cells (VSMC) are normally physiologically quiescent, arranged circumferentially in one or more layers within blood vessel walls. Remodelling of native VSMC to a proliferative state for vascular development, adaptation or repair is driven by platelet-derived growth factor (PDGF). A key effector downstream of PDGF receptors is store-operated calcium entry (SOCE) mediated through the plasma membrane calcium ion channel, ORAI1, which is activated by the endoplasmic reticulum (ER) calcium store sensor, stromal interaction molecule-1 (STIM1). This SOCE was shown to play fundamental roles in the pathological remodelling of VSMC. Exciting transgenic lineage-tracing studies have revealed that the contribution of the phenotypically-modulated VSMC in atherosclerotic plaque formation is more significant than previously appreciated, and growing evidence supports the relevance of ORAI1 signalling in this pathologic remodelling. ORAI1 has also emerged as an attractive potential therapeutic target as it is accessible to extracellular compound inhibition. This is further supported by the progression of several ORAI1 inhibitors into clinical trials. Here we discuss the current knowledge of ORAI1-mediated signalling in pathologic vascular remodelling, particularly in the settings of atherosclerotic cardiovascular diseases (CVDs) and neointimal hyperplasia, and the recent developments in our understanding of the mechanisms by which ORAI1 coordinates VSMC phenotypic remodelling, through the activation of key transcription factor, nuclear factor of activated T-cell (NFAT). In addition, we discuss advances in therapeutic strategies aimed at the ORAI1 target.

## Introduction

Cardiovascular disease (CVD) defines the conditions affecting the heart and blood vessels. CVD is currently the leading cause of global mortality, accounting for an estimated 17 million deaths annually (WHO, 2017). This figure is anticipated to rise as the prevalence in low and middle-income countries increases. CVD is associated with classical risk factors, including obesity (Poirier et al., 2006), smoking (Keto et al., 2016), family history (Dorairaj and Panniyammakal, 2012; Jeemon et al., 2017), and diabetes (Rydén et al., 2013; Shah et al., 2015). Atherosclerotic CVD (e.g., ischaemic heart disease, peripheral arterial disease, cerebrovascular disease, renovascular disease), pulmonary hypertension and aneurysm formation have all been associated with pathological remodelling behaviour of the native vascular smooth muscle cells (VSMC) within the arterial wall. Similarly, failure of surgical revascularisation to treat atherosclerotic CVD lesions (bypass grafting with autologous vein or prosthetic graft) or percutaneous coronary intervention/peripheral artery endovascular intervention (angioplasty+/−stenting) is associated with neointimal hyperplasia (NIH) which is also a manifestation of pathological vascular remodelling. The ability to selectively inhibit such pathological remodelling of VSMC is therefore considered to be a potentially fruitful therapeutic strategy across this range of cardiovascular pathologies. In order to achieve this, an identifiable, specific, druggable target is required. In this review we present an update on the evidence supporting the ORAI1 Ca^2+^ channel as a potential therapeutic target and the current status of inhibitor development. The focus is on atherosclerotic CVD and NIH as little evidence exists regarding aneurysm disease in this context and we recently reviewed the evidence supporting ORAI1 as a target in pulmonary hypertension (Rode et al., 2018).

### VSMC Phenotypic Switching

The VSMC is a specialised cell type which is optimised for vascular contractility and the modulation of vascular tone via its contractile apparatus, which depends on smooth muscle contractile proteins such as alpha smooth muscle actin (α-SMA), smooth muscle myosin heavy chain (SM-MHC) and smooth muscle 22 alpha (SM22α). These “contractile” VSMCs usually reside in the tunica media of the vessel wall and are classically associated with diseases arising from altered vascular tone, such as hypertension. VSMC intracellular free calcium levels and L-type Ca^2+^ channel activity are hallmarks of excessive vascular contractility and are targeted by calcium channel blocking anti-hypertensive drugs in the clinic.

When new blood vessels form during embryogenesis, the local VSMC sub-populations envelop the angiogenic endothelial cells to build the vascular tree. Unlike other specialised cell types, such as the cardiomyocyte, VSMCs are not terminally differentiated. In cases of vascular injury, the contractile VSMCs retain the ability to de-differentiate to an immature, plastic, secretory, and “synthetic” state. These phenotypically modulated VSMCs have reduced expression of contractile proteins, while displaying high indices of proliferation, migration, synthesis and secretion of cytokines and tropoelastins. This phenotypic switch generates a range of de-differentiated VSMC phenotypes, including macrophage-like, osteoblast-like and myofibroblast-like VSMC phenotypes, as reviewed in Sorokin et al. (2020). This heterogeneity in VSMC populations within the healthy vessel wall was evident in the heterogeneity of the single VSMCs transcriptional profiles defined by single cell RNA-sequencing (Dobnikar et al., 2018).

The critical driver of this process is platelet derived growth factor BB (PDGF-BB) signalling through the PDGF receptor beta, PDGFRβ (Owens et al., 2004; Thomas et al., 2009). *In vitro* and *in vivo* studies have shown that PDGF-BB negatively regulates expression of VSMC contractile markers and promotes the phenotypic switch toward a plastic and secretory phenotype. Production of PDGF-BB by activated platelets, macrophages, endothelial cells and even phenotypically modulated VSMCs themselves has been described in atherosclerosis and post-surgical NIH mouse models. This results in downregulation of VSMC contractile markers, and subsequent stimulation of VSMC proliferation and migration, reviewed in Owens et al. (2004). There is evidence to support the concept that as the de-differentiated VSMC lay down new elastin and re-populate the vessel, the elastin itself drives the VSMC back toward their contractile phenotype. This is evidenced by the synthetic behaviour of VSMC obtained from elastin-deficient mice and the reduced NIH observed after elastin delivery to the vessel in porcine model of CVD (Karnik et al., 2003). It has been argued by Owens and others that this is likely to be an evolutionarily conserved mechanism for repairing vascular trauma that conferred a survival advantage to early man. Major trauma is not the main driver of vascular injury in developed societies; rather the risk factors driving the development of CVD cause much less severe but sustained injury to our vasculature. Therefore, VSMC phenotypic modulation becomes sustained and the vascular remodelling response itself becomes part of the pathological process.

### Pathological Vascular Remodelling in Atherosclerosis and NIH

Atherosclerosis is associated with pathological intimal thickening, neovascularisation, and lipoprotein depositions (Virmani et al., 2000). Phenotypically-modulated VSMCs in atherosclerosis have low expression of VSMC contractile markers, and a heightened ability to proliferate and migrate. Synthetic VSMCs have also been associated with increased secretory activities and increased production of extra-cellular matrix (ECM) components, which contribute to the intimal thickening and atherosclerosis progression (Okada et al., 1993; Andreeva et al., 1997). It was long assumed that the role of VSMC was rather limited in atherosclerotic plaque formation. These assumptions were based on conventional VSMC identification approaches; low levels of antibody staining for “classical” VSMC markers, such as α-SMA and SM-MHC were observed in the atherosclerotic plaque. However, as discussed above, the classical VSMC markers are downregulated in phenotypically-modulated VSMC therefore potentially rendering them undetectable via this approach. Recent transgenic lineage-tracing studies have enabled fate tracking of VSMC even following remodelling and loss of contractile protein expression. These elegant studies demonstrated that more than 80% of cells within lesions are phenotypically-modulated VSMC that lack VSMC identification markers (Shankman et al., 2015). Compelling evidence supports the adverse effect of VSMC remodelling to macrophage-like foam cells in lesion pathogenesis. This was demonstrated in a study from the Owens laboratory that utilised an SMC lineage-tracing murine model to study the impact of SMC-specific deletion of the pluripotency factor, Krüppel-like factor 4 (Klf4), which is crucial for the PDGF-induced VSMC phenotypic switch, on atherosclerotic plaque development (Deaton et al., 2009). They showed that loss of Klf4 in VSMC was associated with reduced plaque formation, and improved plaque stability (Shankman et al., 2015), highlighting the key role of VSMC remodelling in the pathogenesis of atherosclerosis and the potential beneficial outcome of inhibiting the extrinsic phenotypic switch to target atherosclerosis. Critically, the same group have demonstrated that VSMC remodelling mediated by the transcription factor Octamer-Binding Protein 4 (Oct4) is crucial for plaque stability, and that Oct4 conditional deletion in VSMC had adverse effects on lesion pathogenesis as shown by the reduced VSMC content in the protective fibrous cap, implying that VSMC phenotypic switching could be beneficial in maintaining plaque stability, and preventing rupture (Otsuka et al., 2015). Additionally, VSMC apoptosis was also shown to be a key feature of plaque vulnerability (Clarke and Bennett, 2006).

The VSMC phenotypic switch is also a key player in the development of NIH. Post-coronary intervention patients remain at risk of developing NIH, with even greater risk in patients with comorbidities, including diabetes and obesity (Silber et al., 2013). Drug-eluting stents that target VSMC proliferation have been shown to improve the clinical outcome following bypass surgery, percutaneous coronary angioplasty, and peripheral revascularisation (Moses et al., 2003), yet a recent systematic review and meta-analysis suggested increased mortality risk 5 years following femoropopliteal application of drug-coated balloons and stents (Katsanos et al., 2018). Despite the beneficial effects of the drug-coated stents and balloons in preventing NIH following angioplasty, the reported possible deleterious long-term side effects of these devices raised concerns about their safety and emphasised the clinical need for new effective therapeutic strategies.

Taken together, strong evidence supports the crucial role of VSMC in the pathophysiology of atherosclerosis, and NIH. VSMC could be directed toward either beneficial or unfavourable remodelling during lesion development. Nevertheless, suitable (and druggable) molecular therapeutic targets to control VSMC phenotypic remodelling remain elusive.

### Store-Operated Calcium Entry (SOCE)

Calcium (Ca^2+^) is a universal second messenger and signalling ion that is crucial for a wide range of cellular processes with a temporal range from milliseconds (e.g., contraction) to hours/days (e.g., gene transcription). The concentration of global as well as compartmentalised Ca^2+^ within the cell regulates Ca^2+^-dependent regulatory pathways that define VSMC function and phenotype, cytoskeletal remodelling and cell proliferation (Beech, 2007). Cytosolic Ca^2+^ influx occurs through plasmalemmal Ca^2+^ channels, including voltage-gated and receptor-operated Ca^2+^ channels, that allow modulated extracellular Ca^2+^ influx into the cell. Additionally, Ca^2+^ fluxes out of and into the major intracellular Ca^2+^ store, the endoplasmic reticulum (ER), are controlled by ryanodine receptors (RyR) and inositol trisphosphate receptors (IP_3_R) on the ER membrane. PDGF-BB, which drives VSMC phenotypic switch, triggers a global rise in intracellular Ca^2+^ through the binding of PDGF-BB to its receptor, PDGFRβ, initiating phosphorylation of the PDGF receptor tyrosine kinase residues, leading to activation of number of signalling pathways implicated in proliferative vascular diseases, including phospholipase C (PLC) and phosphatidylinositol 3-kinase (PI3K). The activated PLC enzymes generate IP_3_, which in turn promotes the activation of IP_3_R and the release of Ca^2+^ waves from the ER to the cytosol.

The phenomenon of extracellular Ca^2+^ influx following intracellular Ca^2+^ release was first observed by Putney in 1977 (Putney, 1977) and formalised into the theory of capacitative Ca^2+^ entry in 1986 (Putney, 1986). The proposed hypothesis was then verified by the observed increase in Ca^2+^ influx in parotid acinar cells following stimulation of store depletion using the sarco/endoplasmic reticulum Ca^2+^-ATPase (SERCA) pump inhibitor, thapsigargin (Takemura et al., 1989). Afterward, via whole-cell patch-clamp analysis in mast cells, Ca^2+^ current following store depletion was recorded and characterised as Ca^2+^-selective inwardly rectifying current, which was termed calcium-release-activated-calcium current (I_CRAC_) (Hoth and Penner, 1992). Today the process of extracellular Ca^2+^ influx upon depletion of the intracellular stores is most commonly described as store-operated Ca^2+^ entry or SOCE and throughout this review we will use this term.

**FIGURE 1:**
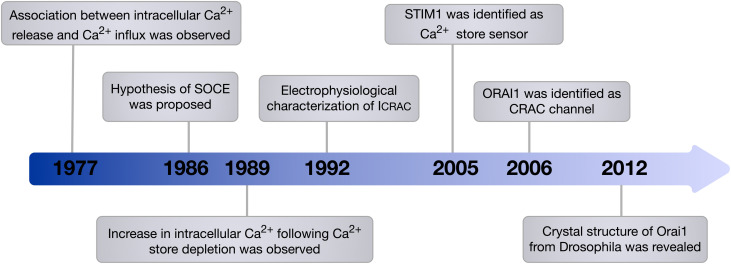
Timeline of the main milestones in the discovery of SOCE. In 1986, Putney proposed the hypothesis of SOCE. This hypothesis was then verified and the components of SOCE, which are STIM1 the Ca^2+^ store sensor and ORAI1 the *de facto* pore-forming subunit of CRAC channel, were identified. Since this discovery, the involvement of ORAI1 in various vital cellular processes and its contribution to diverse diseases have been recognised.

Despite the physiological phenomenon of SOCE being well established, the molecular machinery encoding SOCE remained elusive for many years. In 2005, STIM1 was identified as the ER membrane Ca^2+^ store sensor and a key component of SOCE (Liou et al., 2005; Roos et al., 2005). It has since been demonstrated that a small pool of STIM1 also exists in the plasma membrane (Li et al., 2015). Identification of patients who presented with severe combined immune deficiency (SCID) due to impairment of SOCE in T cells was a key discovery that provided a chance to pinpoint genes encoding calcium-release-activated-calcium current (CRAC) channels (Feske et al., 2005). ORAI1, which is also known as calcium release-activated calcium modulator 1 (CRACM1) and transmembrane protein 142A (TMEM142A) was identified in 2006 by Feske et al. (2006) through a genome-wide screen of linkage in SCID patients that identified a missense mutation in ORAI1, which resulted in impaired I_CRAC_ in T cells, as well as a genome-wide RNA interference screen in *Drosophila melanogaster* which provided support that ORAI1 is the key component of CRAC channels (Figure 1). In addition, genome-wide RNA-interference screens have identified ORAI1 as a key component of SOCE in *D. melanogaster* S2 cells, and confirmed the requirement of ORAI1 for generation of I_CRAC_ (Zhang et al., 2006). Ectopic co-expression of ORAI1 and STIM1 was able to augment SOCE in human embryonic kidney cells (HEK293) and Jurkat T cells, implying their independent role in generating I_CRAC_ (Peinelt et al., 2006). Furthermore, Prakriya et al. (2006) revealed that ORAI1 is the *de facto* pore-forming subunit of CRAC channels, demonstrating that ORAI1 is located at the cell surface, and that mutations within ORAI1 alter the properties of the CRAC current.

It was long thought that SOCE was mediated by members of the transient receptor potential (TRP) channel superfamily, mainly canonical TRP (TRPC) channels, most commonly, TRPC1. Attenuation of SOCE upon TRPC1 knockdown in human submandibular gland (HSG) cells (Liu et al., 2003), and in salivary gland acinar cells isolated from TRPC1 knockout mice (Liu et al., 2007), as well as the augmentation of Ca^2+^ influx following thapsigargin-mediated store-depletion in cells expressing TRPC1 (Zhu et al., 1996; Liu et al., 2003) provided support for this view. Induced expression of mutant TRPC1 that either encodes truncated protein or harbours mutations at the negatively charged residues in the pore-forming region resulted in remarkable reduction of SOCE, implying that TRPC1 could be a molecular component of CRAC channels (Liu et al., 2003).

The discovery of ORAI1 as the *de facto* CRAC channel created debate regarding TRPC1 as a direct component of CRAC channels or indirect modulator of SOCE. ORAI1 was shown to associate with TRPC1, with enhanced interaction upon store depletion, suggesting the involvement of ORAI1/TRPC1 channel in SOCE (Jardin et al., 2008). Induced interaction between ORAI1 and TRPC1 was observed in murine pulmonary arterial smooth muscle cells with elevated SOCE in response to acute hypoxia (Ng et al., 2012). Ávila-Medina et al. (2016) showed via an *in situ* proximity ligation assay that the three channels, ORAI1, TRPC1 and voltage-gated Ca^2+^ channel (Ca_V_1.2) are localised in close proximity in VSMC, suggesting possible functional interactions to modulate Ca^2+^ signalling. Electrostatic interaction between TRPC1 and STIM1 was reported to be essential for TRPC1 channel gating (Zeng et al., 2008). Moreover, inhibition of the interaction between ORAI1 and STIM1, using an antibody directed against the C-terminal region of ORAI1, impaired STIM1 and TRPC1 association and altered TRPC1 function from a store-operated channel into a store-independent (receptor-operated) channel in human platelet cells (Jardin et al., 2008).

Arguing against the role of TRPC1 as a CRAC channel, the activity of TRPC1 was reported to be independent of the ER Ca^2+^ sensor STIM1, as shown by the lack of effect of STIM1 silencing or overexpression on the channel’s activity in HEK293 cells (Wayne et al., 2009). Nonetheless, an alternative model of communication with intracellular stores was suggested by an enhanced TRPC1 interaction with the IP_3_ receptor upon store depletion in human platelets, implying that the role of TRPC1 could include coupling to the IP_3_ receptor in the ER (Rosado et al., 2002). This model was again challenged by the observation that TRPC1 is localised on the intracellular membranes in platelets rather than the plasma membrane (Hassock et al., 2002). It was also reported that TRPC1-induced expression failed to enhance SOCE (Sinkins et al., 1998; Strübing et al., 2001), and VSMC isolated from TRPC1 knockout mice showed no change in SOCE relative to wild type (Dietrich et al., 2007). Despite the evidence supporting the involvement of TRPC1 in SOCE, there is still considerable controversy surrounding the contradictory results of the involvement of TRPC1 in SOCE.

#### STIM1

**FIGURE 2:**
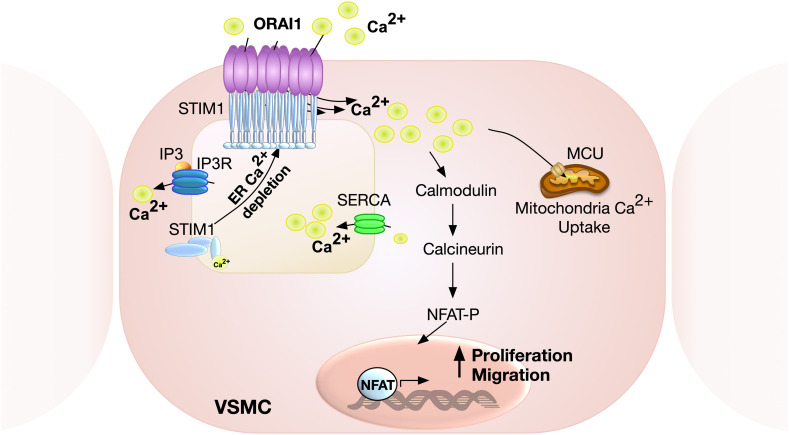
Schematic representation of ORAI1-mediated SOCE in VSMC. Calcium release from ER via inositol 1,4,5-triphosphate (IP_3_) receptor results in store depletion, which induces STIM1 oligomerisation to activate ORAI1 channels. ORAI1-mediated SOCE is associated with activation of nuclear factor of activated T-cell (NFAT), which promotes VSMC proliferation and migration. Ca^2+^ influx via ORAI1 also induces mitochondrial Ca^2+^ uptake via mitochondrial calcium uniporter (MCU).

Live cell imaging and electrophysiology studies have shown that following pharmacological store depletion, STIM1 undergoes oligomerisation, aggregation and translocation to the ER-plasma membrane junctions to activate ORAI1 channels (Wu et al., 2006; Liou et al., 2007; Luik et al., 2008; McKeown et al., 2012). This distribution, however, was not observed in PDGF-mediated ORAI1 activation in VSMC, revealing an alternative non-clustering mechanism of ORAI1 activation that is likely to be more relevant to the physiological setting (McKeown et al., 2012). STIM1 is an ER membrane protein consisting of multiple structural domains, with the N-terminus located in ER lumen encompassing canonical EF-hand, hidden EF-hand, and sterile alpha motif (SAM) domains. The Ca^2+^ sensing ability of STIM1 is mediated by the low Ca^2+^ binding affinity of the canonical EF-hand intra-ER domain. Ca^2+^ depletion and release from the EF-hand domain result in exposure of its hydrophobic residues, EF-SAM monomer transformation into an oligomer, elongation of the C-terminal cytosolic domain, and exposure of its CRAC activation domain (CAD) (Huang et al., 2006; Stathopulos et al., 2006; Covington et al., 2010; Yang et al., 2012b). The ORAI1 channel is subsequently activated by direct interaction with CAD, generating I_CRAC_ (Spassova et al., 2006; Park et al., 2009; Figure 2). STIM1 regulation of Ca^2 +^ signalling is not limited to modulating ORAI1 activity. Other Ca^2+^ channels revealed to be regulated by STIM1 include TRPC1 (Zeng et al., 2008), and the voltage-gated Ca^2+^ channel, Ca_V_1.2 (Wang et al., 2010). Interestingly, while STIM1 activates ORAI1 channels, its interaction with Ca_V_1.2 attenuates the channel’s activity (Wang et al., 2010), supporting a bimodal function of STIM1 in regulating Ca^2+^ signalling by activating SOCE on one hand and inhibiting voltage-gated Ca^2+^ channels on the other.

#### ORAI1 Channel

**FIGURE 3:**
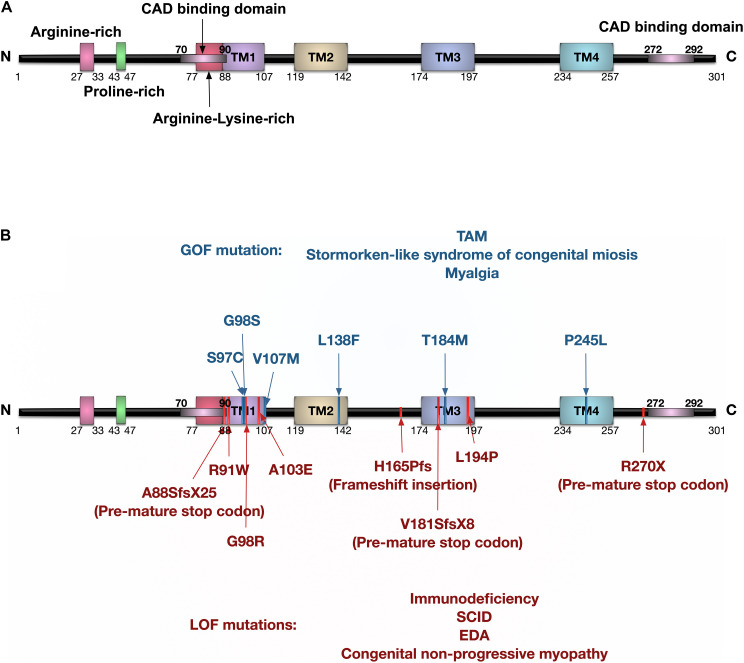
Protein domain structure of human ORAI1 and the reported ORAI1 mutations. **(A)** Human ORAI1 protein comprises four transmembrane (TM) domains, two extracellular loops between TM1-TM2, and between TM3-TM4, one intracellular loop between TM2-TM3, arginine rich domain, proline-rich domain, arginine-lysine rich domain, amino terminus (N), and carboxy terminus (C) containing CRAC activation domain (CAD) binding domains (purple lines). **(B)** The blue lines represent gain-of-function (GOF) single nucleotide polymorphisms (SNPs) reported in tubular aggregate myopathy (TAM) patients with elevated CRAC channel activity. The red lines represent ORAI1 loss-of-function (LOF) mutations reported in patients with immunodeficiency, severe combined immune deficiency (SCID), and ectodermal dysplasia anhydrosis (EDA).

The human *ORAI1* gene is 16.128 Kb in length, located at chromosome 12q24.31 (GRCh38/hg38), and is translated into two protein isoforms: ORAI1α (around 33 KDa) and ORAI1β (around 23 KDa) due to different translation initiation sites (Fukushima et al., 2012). Both ORAI1α and ORAI1β assemble to generate functional CRAC channels, and have similar sub-cellular localisation. Nonetheless, ORAI1β has higher mobility in the plasma membrane as it lacks arginine-rich residues that promote ORAI1α interaction with the phosphatidylinositol-4,5-bisphosphate (PIP_2_) of plasma membrane (Calloway et al., 2011). The ORAI1 protein structure comprises four transmembrane (TM) domains, extracellular TM1-TM2 loop, intracellular TM2-TM3 loop, extracellular TM3-TM4 loop, and cytoplasmic N- and C-termini (Figure 3). ORAI1 was shown to undergo post-translational modifications, including glycosylation of the asparagine 233 residue located at the extracellular loop-2 (Gwack et al., 2007), and phosphorylation of serine residues at positions 27 and 30 (Kawasaki et al., 2010). ORAI1 assembles to form either homomeric or heteromeric channels. Three members of the ORAI family have been identified, with ORAI2 and ORAI3 encoded by separate genes, *ORAI2* and *ORAI3*, respectively. ORAI channels can assemble as hetero-pentameric channels comprising three ORAI1 and two ORAI3 subunits to generate arachidonate-regulated Ca^2+^ (ARC) channel, which is activated independent of store depletion. ARC channels show receptor-mediated activation by intracellular arachidonic acid. Interestingly, only ORAI1α and not ORAI1β participates in the formation of ARC channels (Desai et al., 2015).

ORAI1 monomers are arranged to generate a central ion conduction pore, involving the TM1 domain from each monomer, to generate a circle of TM1 domains. These amino acid residues, surrounding the ion conductance pore, determine the biophysical features of the channel. The glutamic acid residues at position 106 form an outer ring thought to act as a selectivity filter (McNally et al., 2009). Crystal structure determination of the *D. melanogaster* Orai1 revealed assembly as an unusual and unexpected hexameric complex (Hou et al., 2012). However, the data on functional stoichiometry of human ORAI1 (hORAI1) is contradictory. High resolution scanning transmission electron microscopy (STEM) imaging of hORAI1 proteins indicated that they were mainly found as monomers and dimers, with a small fraction found as hexamers (Peckys et al., 2016). Early electrophysiological analysis of hexameric and tetrameric concatemer of hORAI1 suggested that the biophysical properties of the tetrameric concatemer match that of the native CRAC currents, whereas the hexameric concatemer lacked key fingerprint features of CRAC currents (Thompson and Shuttleworth, 2013). More recent electrophysiology studies, however, reported that hexameric hORAI1 concatemer exhibited the key biophysical features of CRAC channels, including Ca^2+^ selectivity, generating unitary current and rapid Ca^2+^ dependent channel inactivation (Yen et al., 2016). Similarly, recent analysis of Ca^2+^ currents mediated by dimeric, trimeric, tetrameric, pentameric, and hexameric concatemers of hORAI1 showed inwardly rectifying store-operated Ca^2+^ current in all oligomeric concatemers similar to that of native CRAC channels (Cai et al., 2016). This study also found that the hORAI1 tetrameric concatemer only contributed with the N-terminal dimer of the construct to generate the channel; unlike the hORAI1 hexamer, in which all subunits contributed to formation of the channel, suggesting that hORAI1 exists in a hexameric configuration (Cai et al., 2016). This is in accord with the atomic force microscopy imaging study that suggested that hORAI1 assembles as a hexamer (Balasuriya et al., 2014). Although several lines of evidence support the idea that hORAI1 channels exist as hexamers, debate remains.

Recent studies have shed the light on the contribution of the different ORAI homologs in the pattern of Ca^2+^ signals and revealed their involvement in Ca^2+^ oscillatory responses (Yoast et al., 2020; Zhang et al., 2020). These oscillations in cytosolic Ca^2+^ concentration, with varying spatiotemporal features, are fundamental cellular signals that are efficiently decoded to activate specific gene transcription and certain cellular responses. The pattern of Ca^2+^ response in cells that lacked either one, two, or the three ORAI homologs was studied and it was shown that ORAI2 and ORAI3 play an essential role in maintaining agonist-induced Ca^2+^ oscillatory responses, while ORAI1 mainly mediates plateaus. These findings suggest that ORAI2 and ORAI3 heteromerisation with ORAI1 plays a role in mediating the channel response to low agonist concentrations, and modulating CRAC channel-mediated gene transcription processes (Yoast et al., 2020).

### ORAI1 in Pathological Vascular Smooth Muscle Cell Remodelling

**FIGURE 4:**
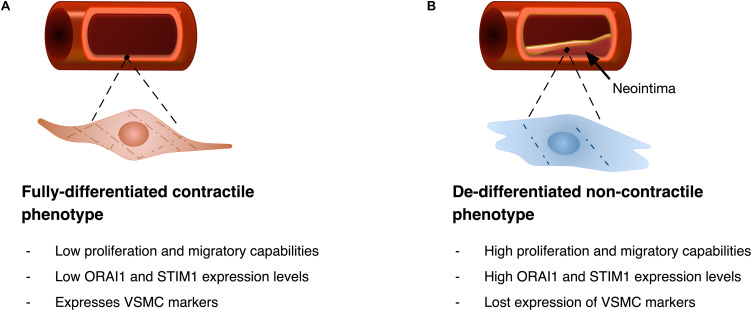
Diagrammatic illustration of VSMC phenotypic remodelling. **(A)** VSMC with fully differentiated contractile phenotype features spindle-like elongated morphology, with low proliferation and migratory abilities, and typically shows low ORAI1 and STIM1 expression levels. Fully differentiated cells express VSMC markers including alpha smooth muscle actin (αSMA), and gamma smooth muscle actin (γSMA). **(B)** Upon phenotypic remodelling VSMC with synthetic phenotype acquires enlarged hypertrophic morphology with high proliferation and migratory abilities. Synthetic VSMCs show high ORAI1 and STIM1 expression levels and low expression of αSMA and γSMA VSMC markers.

Contractile VSMC exhibit almost no proliferation and low expression of ORAI1 and STIM1 proteins. Increased expression of these proteins was shown to be associated with VSMC de-differentiation and remodelling (i.e., the synthetic phenotype, Figure 4). In proliferating cultures of human saphenous vein VSMC (hVSMC), ORAI1 is abundantly expressed and is crucial for SOCE. Where siRNA-induced *ORAI1* silencing suppressed SOCE, and this reduction of SOCE was rescued by transfecting cells with *ORAI1* cDNA, verifying the role of ORAI1 in SOCE in hVSMC (Li et al., 2011b). Inhibition of SOCE either by siRNA-mediated *ORAI1* silencing or pharmacologically using the potent and selective CRAC channel blocker Synta66 (S66) reduced hVSMC migration, with slight reduction of cell proliferation and no effect on cell viability (Li et al., 2011b). Similarly, in human aortic VSMC cultures, impairment of I_CRAC_ via siRNA-mediated *ORAI1* silencing significantly hampered cell proliferation (Baryshnikov et al., 2009). Although the secretory behaviour of VSMC plays a crucial role in pathologic ECM remodelling, little is known about the role of ORAI1 in ECM production and secretion in VSMCs.

Whilst ORAI1 is upregulated in synthetic and proliferative VSMC, the L-type voltage gated Ca_V_1.2 channel was downregulated, which is a trend thought to contribute to the loss of the VSMC contractile function in synthetic phenotype (Gollasch et al., 1998; Ihara et al., 2002). Ca_V_1.2 channel blockers in VSMC promote STIM1-induced ORAI1 activation through promoting STIM1 re-localisation to the ER-PM junctions, in Ca_V_1.2 and store-depletion independent fashion, leading to induced VSMC remodelling (Johnson et al., 2020). These findings not only support the implication of ORAI1/STIM1 signalling in VSMC remodelling but also imply the risk of potential aggravation of vascular remodelling by the Ca_V_1.2 channel blockers, which are routinely used anti-hypertensive medications.

PDGF pro-migratory signalling, an essential component in VSMC phenotypic switching (Yamasaki et al., 2001), was shown to activate ORAI1 channels in hVSMC without inducing ORAI1 redistribution in the plasma membrane (McKeown et al., 2012). In agreement with these findings, Ogawa et al. (2012) showed that PDGF induces pulmonary arterial VSMC proliferation through activation of SOCE. Silencing of either *Orai1* or *Stim1* in cultured rat aortic VSMC disrupted PDGF-induced Ca^2+^ entry and reduced cell migration, verifying that PDGF mediates its effect in VSMC through the ORAI1/STIM1 signalling pathway (Bisaillon et al., 2010). On the other hand, pharmacological potentiation of ORAI1 channel activity using the ORAI1 enhancer (IA65) was shown to promote the PDGF-induced VSMC migration (Azimi et al., 2020). The pro-migratory and proliferative effect of ORAI1 is thought to be mediated through activation of the transcription factor, nuclear factor for activated T cells (NFAT), which potentially promotes the expression of pro-proliferative factors (e.g., IL6, cyclin A, and cyclin D1), as observed from the reduced NFAT nuclear translocation following silencing of *Orai1* or *Stim1* in VSMC (Aubart et al., 2009; Zhang et al., 2011b). *Orai1* silencing in primary VSMC isolated from rabbit aorta resulted in reduced DNA synthesis and cell proliferation (Yang et al., 2012a). In cultured rat synthetic VSMC, knockout of either *Orai1* or *Stim1* reduced cellular proliferative and migratory ability (Potier et al., 2009). Similar results were reported with siRNA-induced silencing of *Stim1* in cultured rat VSMC, resulting in reduced cell proliferation and migration. This effect of *Stim1* silencing on VSMC proliferation and migration was reversed by re-expression of *Stim1* (Guo et al., 2009), demonstrating its vital role in regulating VSMC proliferation. These data highlight the role of ORAI1/STIM1 and SOCE in regulating VSMC switch to a proliferative and migratory phenotype, a process that plays a key role in the aetiology of various vascular pathologies.

In addition to the activation of ORAI1 by PDGF signalling, Urotensin-II-induced VSMC proliferation was mediated through activating SOCE and promoting ORAI1/STIM1 and ORAI1/TRPC interactions (Rodríguez-Moyano et al., 2013). Similar findings were observed by *in vitro* treatment of human coronary VSMC with angiotensin-II, which stimulated VSMC remodelling to proliferative phenotype, and resulted into ORAI1 upregulation leading to augmented SOCE (Liu et al., 2020). Additionally, the angiotensin-II-stimulated human coronary VSMC (Liu et al., 2020) and rat aortic VSMC (Guo et al., 2011) proliferation was hampered by either *ORAI1* or *STIM1* silencing. Sphingosine-1-phosphate (S1P) is another signalling molecule that was shown to induce STIM1 rearrangement, resulting in subsequent activation of ORAI1-mediated SOCE in VSMC. This S1P-triggered activation of SOCE was shown to be higher in synthetic VSMC relative to those with the contractile phenotype, supporting the crucial role ORAI1-mediated SOCE in promoting phenotypic remodelling (Hopson et al., 2011).

The phenotypic modulation of VSMC could lead to their differentiation to an osteoblast-like cells, which is characterised by the expression of osteogenic markers and secretion of calcified matrix, a process known as vascular calcification. This osteogenic differentiation of VSMC is a key player in vascular calcification that can lead to vascular stiffness and atherosclerotic plaque rapture (Durham et al., 2018). ORAI1 has been reported to play a role in this osteogenic reprogramming of human aortic VSMC and vascular calcification. For example, *in vitro* induction of the osteogenic signalling in human aortic VSMC by β-glycerophosphate exposure, as a phosphate donor, or by high extracellular glucose treatment resulted in an upregulation of ORAI1 and STIM1 levels, and this effect on vascular osteogenic signalling was suppressed by ORAI1 silencing or pharmacological inhibition (Ma et al., 2019, 2020; Zhu et al., 2020).

### Evidence Implicating ORAI1 in Pathological Vascular Remodelling in Atherosclerosis and NIH

Mutations in the *ORAI1* gene have been reported in a range of diseases (Figure 3 and Tables 1, 2), including: tubular aggregate myopathy (TAM), SCID, congenital miosis, ectodermal dysplasia anhidrosis (EDA), and Stormorken-like syndrome (Böhm et al., 2017; Garibaldi et al., 2017). The clinical manifestation of ORAI1 deficiency is mainly immunodeficiency, global muscular hypotonia, and defects in dental enamel calcification (Table 1; Feske et al., 2006; McCarl et al., 2009; Badran et al., 2016; Lian et al., 2018). Of interest to the present study however, neo-vascularisation of the cornea was observed in a patient with compound heterozygous missense mutations 308C>A (A103E) and 581T > C (L194P) in the *ORAI1* gene (McCarl et al., 2009). There is also a growing body of evidence linking upregulation of ORAI1 in a wide range of important human diseases from cancer to heart failure. The implication of ORAI1 in human cardiovascular abnormalities was highlighted by the reported association of ORAI1 mutations with Kawasaki disease (KD) susceptibility, which is the leading cause of cardiovascular complications during childhood. These studies have identified rare missense variants in KD patients (Onouchi et al., 2016; Thiha et al., 2019). The reported variants in KD patients, interestingly, include a variant that cause p.Gly98Asp mutation within the TM1 domain that generates the ion conduction pore, which is a mutation known to lead to a constitutive ORAI1 channel activation (Zhang et al., 2011a). These findings signify the potential involvement of ORAI1-mediated signalling in cardiovascular pathologies.

**TABLE 1:** Human ORAI1 (NM_032790.3) mutations and the associated disorders.

**ORAI1 function**	**Mutation**	**Position of mutation**	**Effect on I_CRAC_**	**Disease phenotype**	**References**
Gain-of-function	Heterozygous missense mutation 290C>G (S97C)	TM1	Constitutive activation of I_CRAC_	TAM, congenital miosis	Garibaldi et al., 2017
	Heterozygous missense mutations 292G>A (G98S)	TM1	STIM1-independent constitutive activation of I_CRAC_	TAM, myalgia, occasional mild hypocalcemia, frequent episodes of bleeding from mouth, nose, and bowel	Böhm et al., 2017
	Heterozygous missense mutations 319G>A (V107M)	TM1	STIM1-independent Constitutive activation of I_CRAC_	TAM, myalgia	Böhm et al., 2017
	Heterozygous missense mutations 551C>T (T184M)	TM3	Constitutive activation of I_CRAC_	Mild general weakness, myalgia, hypereosinophilia, pectus excavatum, arched palate, asymptomatic hyperCKemia	Böhm et al., 2017
	Heterozygous missense mutation 292G>A (G98S)	TM1	Constitutive activation of I_CRAC_	TAM, slowly progressive diffuse muscle weakness, hypocalcemia	Endo et al., 2014
	Heterozygous missense mutation 412C>T (L138F)	TM2	Constitutive activation of I_CRAC_	TAM, slowly progressive diffuse muscle weakness	Endo et al., 2014
	Heterozygous missense mutation 734C>T (P245L)	TM4	Prolonged I_CRAC_ activation and reduced inactivation relative to WT	TAM, stormorken-like syndrome of congenital miosis	Nesin et al., 2014
Loss-of-Function	Homozygous missense mutation 271C>T (R91W)	TM1	Defects in SOCE and I_CRAC_	Hereditary SCID, EDA, congenital non-progressive myopathy	Feske et al., 2006
	Homozygous non-sense mutation resulting from frameshift insertion (258_259insA)	Premature termination (A88SfsX25) at the end of TM1	Defects in SOCE and I_CRAC_	SCID due to proliferation defects in T-cells, global muscular hypotonia, defects in dental enamel calcification	McCarl et al., 2009
	Compound heterozygous for two missense mutations 308C>A (A103E) and 581T>C (L194P)	TM1 and TM3 pore-domains	Defects in SOCE and I_CRAC_	SCID, global muscular hypotonia, defects in dental enamel calcification, chronic pulmonary disease due to respiratory muscle insufficiency, eczema, neo-vascularisation of cornea, EDA	McCarl et al., 2009
	Homozygous for missense mutation 581T>C (L194P)	TM3	Defects in SOCE and I_CRAC_	Immunodeficiency, anemia, thrombocytopenia, congenital muscular hypotonia, anhidrosis	Lian et al., 2018
	Homozygous missense mutation 808C>T (R270X) resulting in pre-mature stop codon	C-terminally truncation	Defects in SOCE and I_CRAC_	Immunodeficiency due to proliferation defects in T-cells	Badran et al., 2016
	Homozygous missense mutation, resulting from frameshift insertion 493_494insC (H165Pfs)	C-terminally truncation	Reduced but not abolished I_CRAC_	Immunodeficiency, with normal T-cell numbers and proliferation	Chou et al., 2015
	Homozygous missense mutation 292G>C (G98R)	TM1	Defects in SOCE and I_CRAC_	Immunodeficiency, autoimmune haemolytic anemia, thrombocytopenia, anhidrosis, congenital muscular hypotonia	Lian et al., 2018
	Homozygous for single nucleotide deletion resulting in frameshift mutation (del541C)	Premature termination (V181SfsX8) within TM3	Defects in SOCE and I_CRAC_	Immunodeficiency, reduced T-cell proliferation, muscular hypotonia, EDA	Lian et al., 2018

**I_CRAC_, calcium-release-activated-calcium current; SOCE, store-operated calcium entry; TM, transmembrane; TAM, tubular aggregate myopathy; SCID, severe combined immune deficiency; EDA, ectodermal dysplasia anhidrosis.**

**TABLE 2:** ORAI1 mutations and resulting phenotype in animal models.

**Mutation**	**Position of mutation**	**Model organism**	**Disease phenotype**	**References**
Global *Orai1* knockout in C57BL/6 background	Exon-1 deletion	Mice	Perinatal lethality	Gwack et al., 2008
Global amorphic mutation	ORAI1^R93W/R93W^ in TM1 pore-domain	Mice	Perinatal lethality	Bergmeier et al., 2009; Maus et al., 2017
Global *Orai1* knockout in mixed ICR background	Exon-1 deletion	Mice	Small size, eyelid irritation, and sporadic hair loss; and impaired B-cell proliferation and decreased cytokine production	Gwack et al., 2008
			Small size, reduced and irregular enamel deposition, deficient multinucleated osteoclasts, decreased bone mineral resorption and bone volume, impaired osteoblast differentiation	Robinson et al., 2012
Global *Orai1*^–/–^ via gene trap technology	Deletion of exon-2 and -3	Mice	Small size, defects in integrin activation, degranulation, decreased cytokine production, and defects in the *in vivo* allergic response, no defects in T cell proliferation and differentiation	Vig et al., 2008
Chimeric mice expressing amorphic mutant ORAI1 protein only in blood cells	ORAI1^R93W/R93W^ in TM1 pore-domain	Mice	Defects in platelet integrin activation, degranulation, and surface phosphatidylserine exposure	Bergmeier et al., 2009
Global *Orai1* knockout in mixed ICR background	Deletion of exon-2 and -3	Mice	Osteopenia, decreased bone mineral density and bone volume, despite normal osteoblast differentiation	Hwang et al., 2012
Global *Orai1* deficiency by injection of *Orai1* morpholinos	Targeting splice donor site of exon1 or translational start site	Zebrafish	Muscle weakness, severe heart failure, bradycardia, despite normal cardiomyocyte differentiation	Volkers et al., 2012
Brain- specific *Orai1* deletion	Deletion of exon-2 and -3	Mice	Diminished proliferation of adult neural progenitor cell	Somasundaram et al., 2014
T cell- specific *Orai1* deletion	*Orai1* deletion	Mouse model of EAE	Inhibition of pro-inflammatory cytokines production, and reduced EAE severity	Kaufmann et al., 2016
Global *Orai1* knockout *Orai1*^–/–^ in mixed ICR background	*Orai1* deletion	Mice	Sterile males, severe defects in spermatogenesis, and in elongating spermatid development	Davis et al., 2016
Ectodermal tissues -specific *Orai1* deletion	Deletion of exon-2 and -3	Mice	Impairment of sweat secretion, despite of normal development of sweat glands.	Concepcion et al., 2016
T cell- specific *Orai1/Orai2* double deficient mice	*Orai2* null, *Orai1* T cell-specific knockout	Mice	Impaired T-cell dependent immune response	Vaeth et al., 2017
Pancreatic acinar cell- specific, tamoxifen-inducible *Orai1* deletion	Deletion of exon-2 and -3	Mice	Lack of antimicrobial secretions from pancreatic acinar cells, resulting in intestinal bacterial outgrowth with dysbiosis and increased mortality	Ahuja et al., 2017

**TM, transmembrane; EAE, experimental autoimmune encephalomyelitis.**

#### ORAI1 in the Pathological VSMC Remodelling in NIH and Re-Stenosis

The key role of ORAI1/STIM1 signalling in promoting migration and proliferation of cultured VSMC was supported by *in vivo* studies, where STIM1 (Aubart et al., 2009; Guo et al., 2009) and ORAI1 (Bisaillon et al., 2010) levels were elevated in injured rat carotid arteries following balloon angioplasty, relative to uninjured arteries. Similar elevation of ORAI1 and STIM1 was observed in the intimal hyperplastic lesion in mouse carotid arteries following carotid ligation (Zhang et al., 2011b), and an increased expression of ORAI1 and STIM1 in neointimal VSMC was associated with elevated expression of proliferation markers (Zhang et al., 2011b). Formation of NIH following balloon angioplasty and the expression level of proliferation markers were significantly reduced by either *Stim1* knockdown (Aubart et al., 2009; Guo et al., 2009) or *Orai1* knockdown (Zhang et al., 2011b) induced by viral delivery of siRNA or short-hairpin RNA (shRNA) in rats, respectively, supporting the *in vivo* role of ORAI1 and STIM1 in the pathogenesis of NIH.

It was also shown that angiotensin-II, which is a driver of VSMC remodelling, promotes ORAI1 and STIM1 expression in rat carotid arteries after balloon angioplasty, and that silencing of either *Orai1* or *Stim1* reduced the angiotensin-II-promoted NIH formation in injured rat carotids (Guo et al., 2011). In support, Mancarella et al. (2013) studied the role of STIM1 in SMC function via targeted deletion of *Stim1* specifically in murine smooth muscle tissues using Cre-Lox technology. This murine model was carrying the cre-recombinase transgene under the control of the SM22α smooth muscle-specific promoter that enabled the deletion of *Stim1* in the different smooth muscle tissues. This lack of Stim1 in smooth muscle tissues resulted in abnormal development and impaired contractile response in intestinal and vascular smooth muscles. Development of NIH following carotid artery ligation was shown to be significantly reduced in mice with *Stim1* SMC-specific conditional deletion relative to controls (Mancarella et al., 2013). This study emphasises the role of STIM1 in the formation of NIH. Nonetheless, the reported involvement of STIM1 in NIH does not necessarily indicate similar contribution of ORAI1, due to the multiple pathways and channels involving STIM1 activation.

ORAI1 has been shown to directly interact with various proteins implicated in NIH formation, including members of TRPC family. TRPC1, TRPC3, TRPC4, and TRPC6 were shown to have a role in modulating VSMC proliferation and formation of NIH (Kumar et al., 2006; Jia et al., 2017). Nonetheless, little is known about the potential involvement of ORAI1/TRPC complexes in promoting VSMC proliferation. HOMER1 is a scaffolding protein that has been shown to be able to interact with ORAI1 and a number of TRPC channels. ORAI1 interaction with HOMER1 was shown to be enhanced in VSMC following balloon-injury of carotid arteries, relative to uninjured arteries (Jia et al., 2017). Interestingly, *Homer1* silencing in rat aortic proliferative VSMC resulted in reduction of SOCE, VSMC proliferation, and migration, implying the involvement of ORAI1/HOMER1 interaction in modulating VSMC phenotypic remodelling (Jia et al., 2017).

#### ORAI1 in the Pathological VSMC Remodelling in Atherosclerosis

ORAI1 expression was reported to be elevated in atherosclerotic lesions of Apolipoprotein-E null (ApoE^–/–^) mice on a high-cholesterol diet. *In vivo* silencing of *Orai1* using viral delivery of siRNA against *Orai1* or pharmacological inhibition of ORAI1, using an inhibitor with poor specificity, reduced atherosclerotic plaque formation in ApoE^–/–^ mice fed high-cholesterol diet (Liang et al., 2016). ORAI1-mediated SOCE was shown to be essential for the formation of foam cells, which are macrophages loaded with low-density lipoproteins, a critical step in atherogenesis. *Orai1*-knockdown macrophages exhibited remarkable reduction in their ability to bind and uptake lipoproteins, which subsequently reduced foam cell formation, and atherosclerotic plaque formation (Liang et al., 2016).

Inflammation and cytokine secretion are also major components of the development of atherosclerosis and NIH. Inflammation in atherosclerotic lesions is mediated by pro-inflammatory T helper 1 (Th1) cells that secrete inflammatory cytokines, including interferon-γ (IFN-γ) (Frostegard et al., 1999), which in turn induces macrophage activation and promotes intimal thickening through the promotion of growth factor-induced mitogenesis in VSMC (Ferns et al., 1991; Yokota et al., 1992; Tellides et al., 2000). Knockdown of leukotriene-C4 synthase, which produces the pro-inflammatory mediator leukotriene-C4, and activates ORAI1/ORAI3 channels in VSMC, suppressed neointimal formation in balloon-injured rat carotid artery (Zhang et al., 2015). Furthermore, the emerging role of ORAI1 in lipid metabolism suggests potential involvement of ORAI1 in lipid deposition in atherosclerotic plaque. Maus et al. (2017) revealed that the absence of SOCE due to ORAI1 or STIM1/STIM2 disruption resulted in increased deposition of lipid droplets in murine liver, skeletal muscle, and heart muscle. Additionally, TAM patients with ORAI1 loss-of-function p.Gly98Arg mutation showed deposition of lipid droplets in skeletal muscles, and increased lipid content in patient fibroblasts relative to healthy donors, due to impaired lipolysis (Maus et al., 2017). SOCE was shown to modulate the expression of key enzymes involved in the mitochondrial fatty acid oxidation, and regulate the expression of neutral lipases and a number of transcription regulators that modulate lipolysis (Maus et al., 2017). This is in accord with the previously reported reduction of SOCE associated with lipid accumulation in rat liver cells (Wilson et al., 2015). The involvement of ORAI1 in lipid metabolism foreshadows its potential role in pathogenic mechanisms underlying atherosclerosis, including lipid deposition and formation of the fatty streak.

The ORAI1/ORAI3 ARC store-independent channel was reported to be activated in VSMC after thrombin-mediated induced phenotypic remodelling (González-Cobos et al., 2013). Platelet activation contributes to the initiation and progression of atherosclerosis (Methia et al., 2001; Pratico et al., 2001; Massberg et al., 2002) and platelet adhesion to vascular endothelium was observed before the development of atherosclerotic lesions in ApoE^–/–^ mice (Massberg et al., 2002). Defective SOCE in both *Orai1*^–/–^ or *Stim1*^–/–^ mice resulted in impaired platelet activation and thrombus formation (Varga-Szabo et al., 2008; Braun et al., 2009). Similarly, introduction of a SNP in the EF-hand of murine STIM1 impaired its activation in response to ER Ca^2+^ deletion and resulted in macrothrombocytopenia and impaired platelet activation (Grosse et al., 2007). Furthermore, gain-of-function mutations in STIM1 were observed in patients with York Platelet syndrome (Markello et al., 2015) and thrombocytopenia (Nesin et al., 2014). Impaired platelet function was also observed in patients with ORAI1 mutations (Table 1; Nagy et al., 2018). The involvement of ORAI1-mediated SOCE in normal platelet function implies the potential involvement of ORAI1 abnormalities in platelet activation and adhesion in atherosclerosis.

### Pharmacological Modulation of SOCE

The link between SOCE and vascular remodelling rendered CRAC channels as promising therapeutic targets. Newly identified inhibitors of SOCE have increased understanding of the physiological roles of SOCE and emerged as attractive candidates with the potential to enable pharmacological modulation of CRAC channels. Nonetheless, many of these agents have indefinite mechanism of action and those with the specificity to distinguish between different Ca^2+^ channels remains elusive (Table 3).

**TABLE 3:** Features of ORAI1 channel pharmacological inhibitors.

**Compound**	**Reported potential mode of action**	**Other targets**	**Effective concentrations**
*Lanthanides (Gd^3+^ and La^3+^)*	Direct binding to the extracellular loop of ORAI1 channel	Ca_V_1.2 and TRP channels	Effective at low nanomolar concentration 5 μM Gd^3+^ blocked SOCE in rat synthetic VSMC and in A7R5 VSMC line
*2-APB*	Maintain STIM1 at its resting state Direct inhibition to ORAI1	ORAI2, ORAI3, IP_3_ receptor, SERCA pumps, and members of the TRP channel superfamily	IC_50_ of 10 μM in Jurkat T cells, 4.8 μM in IP_3_R-knockout DT-40, 2.9 μM in CHO cells, and 6.5 μM in HeLa cells
*Carboxyamidotriazole (CAI)*	Indirect inhibition, mitochondrial membrane depolarisation	Inhibits non-voltage gated calcium influx	IC_50_ of around 0.5 μM in HEK293 cells
*The 7-azaindole series, compound (14 d)*	Unclear	Unknown	IC_50_ of 150 ± 22 nM in Jurkat T cells
*The indazole-3-carboxamide series, compound (12 d)*	Unclear	Unknown	IC_50_ of 0.67 μM in RBL-2H3 cells
*pyrtriazole series, compound (39)*	Unclear	Selective for SOCE over CaVs, TRPV1 and TRPM8	IC_50_ of 4.4 μM in HEK293 cells
*DPB162-AE*	Reduction of STIM1 clustering upon store depletion Inhibit STIM1/ORAI1 functional coupling	Induce Ca^2+^ leak from the ER	IC_50_ of 27 nM in DT-40 cells, 190 nM in CHO cells, and 620 nM in HeLa cells
*SKF-96365*	Unclear	TRPC, Ca_V_1.2, voltage-gated Na^+^ channels, ATP sensitive K^+^ channels, and ER Ca^2+^ pumps	IC_50_ of 12 μM in Jurkat T cells
*Synta66*	Potentially binds to the extracellular loop 1 and 3 regions of ORAI1	No off-targets have yet been identified	IC_50_ of 3 μM in RBL cells, 1 μM in Jurkat T cell and 26 nM in VSMC
CM4620	Unclear	No off-targets have yet been identified	IC_50_ of ∼0.1 μM in Orai1/STIM1 overexpressing HEK293
*YM-58483*	Unclear	Inhibits TRPC3, and TRPC5 activity Enhances the activity of TRPM4 channels	IC_50_ of 10–100 nM in Jurkat T cells
*GSK7975A*	Altering channel pore geometry	ORAI3, L-type Ca^2+^, and TRPV6 channels	IC_50_ of 4 μM in HEK293 cells
*AnCOA4*	Interaction with the C-terminus of ORAI1 Inhibition STIM1/ORAI1 interaction	Unknown	20 μM AnCoA4 resulted in 80% SOCE inhibition in HEK293 cells
*RO2959*	Unclear	5-hydroxytryptamine receptor 2B (5-HT_2B_) and the peripheral benzodiazepine (BZD) receptors	IC_50_ of 400 nM in RBL cells, 25 nM in CHO cells, and 260 nM in CD4^+^ T cells
*JPIII*	Unclear	No off-targets have yet been identified	IC_50_ of 299 nM in HEK293 cells

**IC_50_, Half-maximal inhibitory concentration; DT-40, Chicken lymphoblast cell line; CHO, Chinese hamster ovary cells, RBL, Rat chemically induced basophils; TRP, transient receptor potential.**

**FIGURE 5:**
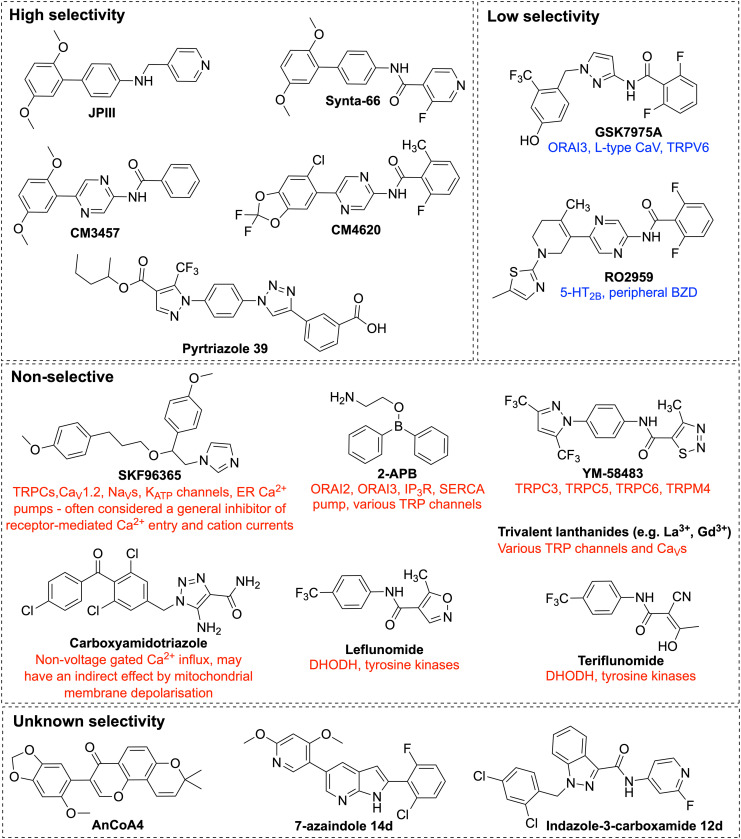
Chemical structures of ORAI1 channel pharmacological inhibitors. Illustration of the chemical structures of ORAI1 channel inhibitors and a list of their previously reported off-targets. The ORAI1 inhibitors were grouped by their reported specificity against ORAI1. 2-APB, 2-Aminoethyldiphenyl Borate; TRP, transient receptor potential; 5-HT_2B_, 5-hydroxytryptamine receptor 2B; BZD, benzodiazepine receptors; DHODH, dihydro-orotate dehydrogenase.

***Lanthanides (Gd^3+^ and La^3+^)*** are widely studied potent inhibitors of CRAC channels, which block SOCE at the nanomolar concentration range. At a concentration of 5 μM, Gd^3+^ was reported to block SOCE in rat synthetic VSMC and in the A7R5 VSMC line (Potier et al., 2009). Sensitivity to inhibition by lanthanides is a key feature of CRAC currents that differentiate CRAC channels from other Ca^2+^ channels. Gd^3+^ is equipotent toward the three ORAI homologs, ORAI1, ORAI2, and ORAI3 (Zhang et al., 2020). Despite the high potency of lanthanides, their efficiency as CRAC channel inhibitors is limited by their high plasma protein binding affinity, low solubility in presence of other multivalent ions, and their limited specificity as CRAC channel inhibitors at higher concentration (Figure 5). At concentrations more than 1 μM, lanthanides inhibit activity of voltage-gated calcium ion channels (Reichling and MacDermott, 1991), and TRPC channels (Halaszovich et al., 2000; Trebak et al., 2002). It is thought that lanthanides exhibit their activity as CRAC channel blockers through binding to acidic residues on the extracellular loop of ORAI1, rather than competing for the Ca^2+^ binding sites within the ion conduction pore of the channel. In support of this theory, the ability of Gd^3+^ to inhibit CRAC channel activity was significantly reduced in cells expressing mutant *ORAI1* gene carrying charge-neutralising mutations of aspartate (D) residues within the TM1-TM2 extracellular loop of ORAI1 (Yeromin et al., 2006). Similarly, McNally et al. (2009) showed that mutations of acidic residues in the human *ORAI1* gene at either of Q108, D110, D112, or D114 of the TM1-TM2 loop reduced the potency of La^3+^ inhibition. However, mutation at position 106, which serves as the ion selectivity filter and is located within TM1 domain that generates the central ion conduction pore, did not affect the ability of La^3+^ to inhibit the channel activity. These observations suggest that lanthanides bind to residues at the extracellular TM1-TM2 loop, rather than binding deeper in the ion conduction pore of ORAI1 channels (McNally et al., 2009).

***Imidazole antimycotic drugs*** including SKF-96365 (Figure 5), econazole and miconazole were shown to inhibit I_CRAC_ in a wide range of cell types. SKF-96365 was first identified in Ca^2+^ signalling studies as an inhibitor of receptor-mediated Ca^2+^ influx, with inhibitory effect in the micromolar range in platelets, neutrophils and endothelial cells (Merritt et al., 1990; Franzius et al., 1994). It was also shown to inhibit SOCE in Jurkat T cells (Chung et al., 1994). In a mouse model of atherosclerosis, *in vivo* administration of SKF-96365 remarkably reduced atherosclerotic plaque development (Liang et al., 2016). Nonetheless, the observed effect is not necessary attributed to SOCE inhibition. A wide spectrum of ion channels were reported to be targeted by SKF-96365, including receptor- and store-operated TRPC channels, voltage gated Ca^2+^ channels (Merritt et al., 1990; Singh et al., 2010), voltage gated sodium channels (Chen et al., 2015), and ATP sensitive potassium channels (K_ATP_) (Tanahashi et al., 2016). Furthermore, earlier studies reported that SKF-96365 not only acts as an ion influx inhibitor, micromolar concentrations of SKF-96365 also inhibited ER Ca^2+^ pumps in human endothelial cells (Iouzalen et al., 1996), and activated reverse operation of Na^+^/Ca^2+^ exchanger (NCX) resulting in Ca^2+^ uptake and sustained intracellular Ca^2+^ elevation in cancer cells (Song et al., 2014). SKF-96365 has served as a powerful tool to characterise mechanisms of Ca^2+^ entry, however, the multiple targets of SKF-96365 could hinder its use as I_CRAC_ inhibitor and limit its translation toward potential therapies. Analogues of SKF-96365 were reported to have higher potency in inhibiting SOCE in B lymphocyte cells; however, further studies are still needed to assess their specificity (Dago et al., 2018).

***Carboxyamidotriazole (CAI)*** (Figure 5) was identified as a Ca^2+^ influx inhibitor, showing antiproliferative and antimetastatic effects in multiple cancer cell lines and *in vivo* xenograft models (Kohn and Liotta, 1990; Hupe et al., 1991; Kohn et al., 1992; Wasilenko et al., 1996; Wu et al., 1997). Its anticancer activity has been linked to inhibition of SOCE, but investigations have been complicated by it being a general inhibitor of non-voltage gated calcium influx (Enfissi et al., 2004). Whilst most research on CAI has focused on its anticancer effects, it has also shown promise in other fields. Through a reduction in pro-inflammatory cytokines, CAI has shown benefits in a rat model of rheumatoid arthritis (Zhu et al., 2015) and a mouse model of inflammatory bowel disease (Guo et al., 2012), although the exact molecular mechanism was not known. There is also much evidence for CAI as an anti-angiogenic agent in cancer (Luzzi et al., 1998; Bauer et al., 2000; Faehling et al., 2002) and retinal neovascularisation (Afzal et al., 2010), although it does not appear to have been investigated for pathological vascular remodelling.

***2-Aminoethyldiphenyl Borate (2-APB)*** is one of the most thoroughly studied SOCE inhibitors (Figure 5). It was initially thought to have inhibitory effect on Ca^2+^ signalling through being a membrane permeable IP_3_ receptor-antagonist (Maruyama et al., 1997; Missiaen et al., 2001), and was also reported to be associated with store-operated Ca^2+^ channel inhibition; nonetheless, this inhibition was interpreted as a potential consequence of IP_3_ receptor inhibition (Ma et al., 2000). Then, Gregory et al. (2001) showed via whole-cell patch-clamp analysis of rat hepatoma cells that 2-APB inhibited inward Ca^2+^ current induced by IP_3_, implying that 2-APB could be acting as a blocker of SOCE. This finding was further supported by the ability of 2-APB to block SOCE in IP_3_ receptor-knockout cells, and the observed more potent inhibition when applied extracellularly relative to intracellularly (Bakowski et al., 2001; Ma et al., 2001; Prakriya and Lewis, 2001). It was reported that this inhibitory effect is mediated via enhancing STIM1 intramolecular interactions, which subsequently maintain STIM1 at its resting state, as well as a potential direct inhibition of ORAI1 as shown by the observed 2-APB inhibition of SOCE in a STIM-independent constitutively active ORAI1 mutant (Wei et al., 2016). Interestingly, 2-APB was shown to exhibit a bimodal effect on SOCE, in which low concentrations (1–5 μM) promote SOCE, while higher ones (more than 10 μM) have a strong inhibitory effect (Prakriya and Lewis, 2001). Besides the inhibition of ORAI1, 2-APB at 50 μM concentration showed weak inhibitory effect of ORAI2, and significantly potentiated the activity of ORAI3 channels in a store-independent manner (Lis et al., 2007; Peinelt et al., 2008). 2-APB was also shown to inhibit SERCA pumps (Bilmen et al., 2002; Peppiatt et al., 2003) and to modulate the activity of members of the TRP channel superfamily, including members of TRPM subfamily, TRPM6, TRPM7 (Li et al., 2006) and TRPM8 (Hu et al., 2004), TRPV subfamily, TRPV1, TRPV2, and TRPV3 (Hu et al., 2004) and members of TRPC subfamily, TRPC3 (Trebak et al., 2002) and TRPC6 (Hu et al., 2004). Its multiple targets and limited specificity promoted the need to develop analogues of 2-APB with improved selectivity and potency. Zhou et al. (2007), assessed the potency of 166 2-APB analogues and two analogues (DPB025 and DPB083) were identified to have higher specificity to SOCE inhibition relative to that of 2-APB. Additionally, analogues including DPB161-AE, DPB163, and DPB162-AE that are around 100-fold more potent than 2-APB itself were identified (Zhou et al., 2007; Goto et al., 2010). It was, however, shown that the effect of DPB162-AE on Ca^2+^ signalling is not limited to inhibition of CRAC channels, but also induced Ca^2+^ leak from the ER resulting in Ca^2+^ store depletion without inhibiting SERCA pumps (Bittremieux et al., 2017). The mechanisms underlying the diverse effects of 2-APB and its analogues on SOCE remain poorly understood and more studies are still need to clarify their mechanism of action. Due to its wide range of protein targets, various clinically relevant biological effects have been observed and recently reviewed, including immunomodulatory, anti-cancer, neuroprotective, and in the GI system (Rosalez et al., 2020). Of relevance to CVD, 2-APB has been studied and shown benefits in models of atherosclerosis (Ewart et al., 2017; Simo-Cheyou et al., 2017), hypertension (Bencze et al., 2015), and vascular calcification (Lee et al., 2020), although these effects have mostly been attributed to its effects on TRP channels or the IP_3_ receptor.

***YM-58483*,** also known as bis(trifluoromethyl)pyrazole-2 (BTP2 or Pyr2) (Figure 5), is a pyrazole derivative that was identified as an I_CRAC_ blocker with an IC_50_ of 10–100 nM in Jurkat T cells (Ishikawa et al., 2003; Zitt et al., 2004). YM-58483-mediated I_CRAC_ inhibition resulted in reduced T cell activation, and proliferation, as well as reduced cytokine production. It exhibits higher specificity in inhibiting CRAC channels over voltage gated Ca^2+^ channels, without off-target effects observed on ER Ca^2+^ pumps or potassium channels. Despite its remarkable potency, its inhibitory effect on I_CRAC_ is limited by the long incubation time that is needed to achieve this high potency, with around 75% inhibition of I_CRAC_ achieved within 2 h of incubation with Jurkat T cells (Zitt et al., 2004). The inhibitory effect of YM-58483 was only observed when applied extracellularly implying that it probably interacts with the extracellular side of CRAC channels (Zitt et al., 2004). In contrast, others suggested that YM-58483 inhibits I_CRAC_ through binding to an actin reorganisation protein, Drebrin, and thus disrupting actin cytoskeleton (Mercer et al., 2010). Besides inhibiting CRAC channels, YM-58483 was shown to promote the activity of TRPM4 channels at low nanomolar concentrations (Takezawa et al., 2006) and inhibit the activity of number of TRPC channels, including TRPC3, and TRPC5 (He et al., 2005). It has mostly been studied for its effects in inflammatory disease, including asthma and rheumatoid arthritis (Yoshino et al., 2007; Miyoshi et al., 2018; Sogkas et al., 2018), as well as pain and neurology (Qi et al., 2016; Orem et al., 2020). Of relevance to vascular pathology, YM-58483 and SKF-96365 have been used to characterise SOCE in models of diabetic vasculopathy, in which SOCE is reduced compared to non-diabetic VSMCs (Schach et al., 2020).

***GSK7975A, GSK5503A, and GSK5498A*** are pyrazole derivatives developed by GlaxoSmithKline for inflammatory and immune disorders. GSK5498A was reported to inhibit I_CRAC_ at IC_50_ of around 1 μM in HEK293 co-expressing STIM1 and ORAI1 (Ashmole et al., 2012). GSK7975A was shown to be effective in models of acute pancreatitis (Gerasimenko et al., 2013; Voronina et al., 2015). GSK7975A and GSK5503A were shown to have similar inhibitory effects on I_CRAC_. Similar to the earlier pyrazole derivative YM-58483, GSK7975A, and GSK5503A showed slow onset of I_CRAC_ inhibition. GSK7975A inhibits both ORAI1 and ORAI3 at similar IC_50_ of around 4 μM in HEK293 cells expressing STIM1, as well as ORAI1 or ORAI3 (Derler et al., 2013). It was also reported that GSK7975A largely inhibits both ORAI1 and ORAI2, with less inhibitory effect against ORAI3 (Zhang et al., 2020). Its inhibitory effect was suggested to be mediated through direct interaction with ORAI1 channel, without affecting STIM1 oligomerisation or ORAI1-STIM1 interactions (Derler et al., 2013). This inhibitory effect was significantly reduced in cells expressing pore mutant ORAI1, relative to those expressing wildtype ORAI1, suggesting mechanism of inhibition via altering the pore geometry of ORAI1 channels (Derler et al., 2013). GSK7975A showed partial inhibition of the activity of L-type Ca^2+^ channels, and blocked TRPV6 channel activity. Failure to differentiate between ORAI1 and ORAI3 channels, as well as inhibition of L-type Ca^2+^, and TRPV6 channels, limit its specificity as an ORAI1 channel blocker.

***Synta66 (S66)*** (Figure 5) is an I_CRAC_ inhibitor showing an inhibitory effect in the micromolar concentration range. A lack of off-target effects on the inwardly rectifying K^+^ current or Ca^2+^ pumps suggested selectivity against I_CRAC_ (Ng et al., 2008). Its remarkable selectivity was then revealed by radioligand binding assays and functional assays which showed that S66 did not affect the activity of wide spectrum of receptors, and ion channels, including voltage-gated Ca^2+^ channels, and Na^+^ channels (Di Sabatino et al., 2009). In support, Li et al. (2011b) showed that S66 did not affect STIM1 aggregation, or the activity of TRPC5 channels, TRPC1/5 channels, or even the store operated non-selective cationic current. S66 showed remarkable inhibition of ORAI1 activity, whereas it showed only minimal activation of ORAI2 and inhibition of ORAI3 in HEK293 cells that lacked the three native ORAI homologs and expressed a specific individual ORAI homolog along with STIM1 (Zhang et al., 2020). Computational docking simulations have shown that S66 potentially binds to the extracellular loop 1 and 3 regions of ORAI1, which are regions at close proximity to the selectivity filter. The inhibitory effect of S66 was also shown to be weakened in cells expressing ORAI1 mutations affecting the channel selectivity. This was demonstrated by the impaired S66-mediated SOCE inhibition by the ORAI1 mutation (E106D) within the region that encodes for the glutamate residues that form the channel selectivity filter. Similar impairment of the S66 inhibitory effect was observed with other ORAI1 mutations that result into non-selective currents, like the mutant ORAI1 that carries mutations within its extracellular loop1 or loop 3 regions (Waldherr et al., 2020). S66 is a potent inhibitor of SOCE in VSMCs isolated from human saphenous veins with an IC_50_ of around 26 nM and resulted in significant reduction of VSMC migration (Li et al., 2011b). It has also been found to inhibit endothelial cell migration and tube formation *in vitro* and angiogenesis *in vivo* (Li et al., 2011a). Interestingly, S66 showed remarkably higher potency in inhibiting I_CRAC_ in VSMC than its reported potency in the RBL cells (Ng et al., 2008), Jurkat T cell (Di Sabatino et al., 2009), and in leucocytes (Li et al., 2011b). The high potency of S66 in VSMC relative to other cell types raises the promise to selectively modulate CRAC channels in VSMC, and to enable targeting vascular remodelling. Nonetheless, the lack of information regarding the mechanisms underlying S66-induced I_CRAC_ inhibition and its poor aqueous solubility render it far from being translated into therapies.

***RO2959*** was developed by Roche as a CRAC channel blocker that is effective in the nanomolar concentration range. It showed higher selectivity for ORAI1 inhibition over ORAI2 and ORAI3. It was screened against a range of ion channels, but showed no off-target effects on voltage-gated Ca^2+^ channels, Na^+^, K^+^ channels, members of TRPC or TRPM channels, indicating specificity in inhibiting ORAI1 channels. It was also screened against cell receptors and transporters and two receptors were considered to be inhibited by RO2959, which are the 5-hydroxytryptamine receptor 2B (5-HT2B) and the peripheral benzodiazepine (BZD) receptors, showing 87 and 89% inhibition, respectively, at 3 μM concentration of RO2959 (Chen et al., 2013). RO2959-mediated I_CRAC_ inhibition in human CD4^+^ T cells resulted in reduction of cell proliferation and cytokine production, signifying the role of I_CRAC_ in T cell function (Chen et al., 2013). Nevertheless, the mechanism of action of RO2959 remains elusive.

***AnCoA4*** was discovered by a commercial small molecule microarray of 12,000 compounds (Sadaghiani et al., 2014). Rather than screening against whole cells, this technique uses minimal functional domains, which are purified isolated domains of ORAI1 and STIM1 known to be vital for SOCE. This is to allow only small molecules that directly bind to ORAI1 or STIM1 to be identified as hits, avoiding those that indirectly affect SOCE. AnCoA4, at 20 μM concentration, showed 80% inhibition of SOCE in HEK293 cells co-expressing STIM1 and ORAI1. A binding site for AnCoA4 on the C-terminus of ORAI1 was proposed, using a fluorescence aggregation assay, surface plasmon resonance and the FRED docking algorithm. AnCoA4 was also found to inhibit the ORAI1/STIM1 interaction, on the C-terminus, and to compete with STIM1 for ORAI1 binding, supporting the binding region prediction (Sadaghiani et al., 2014).

***The 7-azaindole series*** of SOCE inhibitors was developed based on the structures of YM-58483 and Synta-66, a series of 7-azaindole SOCE inhibitors were developed for inflammatory respiratory diseases. Lead compound (14 d) inhibited SOCE with an IC_50_ of 150 ± 22 nM in Jurkat T cells, and its administration in a rat model of allergic respiratory inflammation was associated with a dose-dependent inhibition of eosinophils, showing promise as a therapeutic strategy (Esteve et al., 2015).

***The indazole-3-carboxamide series*** was also developed as anti-inflammatory compounds, and shows moderate SOCE inhibition, with lead compound (12 d) showing dose-dependent inhibition of mast cell activation and pro-inflammatory cytokine release in the range of 0.28–1.60 μM IC_50_ in RBL-2H3 cells (Bai et al., 2017). In this series, the “reverse” amide bond isomers were found to have significantly reduced activity, with most only inhibiting at >100 μM. This effect has not been reported in any other ORAI1 inhibitors, and most of those containing amide bonds are in reverse to 12 d (YM-58483, Synta-66, RO2959, GSK series, CalciMedica series). Compound (12 d) has also been developed into an indole derivative, MCS-01, which inhibits Ca^2+^ influx at 1.6 μM and is being developed as a topical mast cell stabilising treatment to improve diabetic wound healing (Tellechea et al., 2020).

***Leflunomide and teriflunomide*** were identified as weakly potent ORAI1 inhibitors alongside several other FDA-approved drugs (Rahman and Rahman, 2017). Leflunomide is used clinically as a dihydro-orotate dehydrogenase inhibitor to treat rheumatoid and psoriatic arthritis; teriflunomide was later approved for the treatment of multiple sclerosis (Fragoso and Brooks, 2015). These were identified in a virtual ligand-based screen for 3D shape and electrostatics using the structures of Synta66, AnCoA4, YM-58483 and its analogue Pyr6 as bait. Leflunomide and its active metabolite teriflunomide were identified as being able to inhibit SOCE at clinically relevant concentrations, with IC_50_ of around 10 μM for leflunomide and 21 μM for teriflunomide.

***CM4620*** is a small-molecule ORAI1 inhibitor developed by CalciMedica, which has shown potent inhibition of ORAI1 activity with IC_50_ of 119 nM and a less potent effect against ORAI2 channels with an IC_50_ of 895 nM (CalciMedica, 2016). CM4620 was showed to be effective in reducing the severity of acute pancreatitis in pre-clinical models (Waldron et al., 2019), and reached clinical trials for acute pancreatitis and is currently being tested in Phase II clinical trials for use in patients with severe COVID-19 pneumonia (Miller et al., 2020). These ongoing clinical trials emphasise the efficacy, safety, and tolerability of ORAI1 inhibitors in patients and highlights the promising potential of using ORAI1 inhibition as new therapies. An older compound, CM3457, has shown inhibition of various interleukins and other immune functions in different cell lines (Ramos et al., 2012). It was also shown to be selective over a small panel of potassium, sodium and calcium channels, mostly those involved in cardiac function, and is effective in rat models of arthritis and asthma (Ramos et al., 2012).

***The pyrtriazole series*** has been developed as an anti-inflammatory SOCE inhibitors based on the structures of the Pyr family of compounds. Lead Pyrtriazole compound (39) showed an IC_50_ of 4.4 μM for SOCE inhibition in HEK293 cells and was reported to be selective for SOCE over voltage gated Ca^2+^ channels, TRPV1 and TRPM8, although an analogue was found to activate TRPV1. Pyrtriazole 39 was taken into a mouse model of acute pancreatitis and found to reduce oedema, inflammation and apoptosis, all hallmarks of pancreatitis. This series also contains two compounds which were unexpectedly found to be SOCE activators, activating the channel at 198–236% entry and 142–197% entry at 10 μM in three different cell lines (Riva et al., 2018).

***Rhizen Pharmaceuticals*** have developed inhibitors of SOCE for the treatment of cancers, two of which have reached clinical trials. RP3128 is orally active and effective in a guinea pig model of asthma (Vakkalanka et al., 2013; Sutovska et al., 2016), and was taken into a Phase I dose escalation safety study (Barde et al., 2020). Another compound, RP4010, has been investigated for esophageal squamous cell cancer and is potent and effective in several cancer cell lines and in xenograft mouse models (Cui et al., 2018). It required around 2 h to demonstrate maximal inhibition of SOCE, and so may have an indirect effect on the channel rather than directly blocking ORAI1 (Cui et al., 2018). It was entered into Phase I safety studies for the treatment of relapsed non-Hodgkin’s lymphoma, but the trial has been terminated, because of pharmacokinetic (PK) and safety reasons (US National Library of Medicine, 2017).

***JPIII (4-(2,5-dimethoxyphenyl)-N-[(pyridin-4-yl)methyl] aniline)*** is a novel analogue of S66 that we have recently identified as a potent ORAI1 inhibitor with sufficiently improved pharmacokinetics compared to S66 (Bartoli et al., 2020). JPIII showed potent inhibitory effects of SOCE at the nanomolar range, with IC_50_ of 399 nM in HEK293 cells. Besides its potency, JPIII also showed remarkable selectivity against ORAI1, and did not affect the activity of ORAI3, TRPC5, TRPC6, TRPC4, TRPC5, TRPM2, or hERG channels. It also showed high efficacy, without any obvious side-effects when administered *in vivo* in murine models (Bartoli et al., 2020). The high potency at the target, the selectivity, the improved pharmacokinetics compared to S66, which is limited by its poor aqueous solubility, as well as the *in vivo* safety and efficacy in pre-clinical models highlight the great potential of JPIII to be used as an *in vivo* tool to study the effects of ORAI1 inhibition on VSMC biology. It is, however, limited by lack of information about the mechanism by which it inhibits ORAI1 activity.

These ORAI1 channel inhibitors are valuable tools to study the role of SOCE in health and disease, and paves the way for the development of therapeutic ORAI1 inhibitors that target pathologic remodelling. The reported implications of ORAI1-mediated signalling in VSMC phenotypic switching and in vascular pathologies highlight the therapeutic promise of ORAI1-targeted approaches. This is further supported by the role of ORAI1 in immune cell function, inflammation, and lipid homeostasis, which are key components in the development of atherosclerosis and NIH. A number of pharmacological inhibitors have now reached clinical trials for severe plaque psoriasis, acute pancreatitis, asthma and coronavirus disease 2019 (COVID-19)-associated severe pneumonia (Stauderman, 2018; Miller et al., 2020), which highlights the therapeutic potential of the ORAI1 channel inhibitors and the tolerability of the ORAI1 inhibitors in patients. Activators of ORAI1 activity, as shown in the recently developed enhancer of ORAI1 activity, IA65 (Azimi et al., 2020), could also be useful tools to help further define the role of ORAI1 in pathophysiology.

Understanding the nature of ORAI1 involvement in health and diseases holds promise to allow fine-tuning of VSMC phenotypic remodelling to its normal physiological levels. The nature of ORAI1 involvement in vascular development, angiogenesis, vascular physiology and vascular diseases is still unclear. This could be attributed to lack of information regarding ORAI1 dysregulation in adults, because of the early mortality associated with ORAI1 mutations, as well as the perinatal lethality of ORAI1 deletion in animal models. Further studies are still needed to elucidate the role of ORAI1 in vascular development, physiology and diseases, as well as the *in vivo* consequences that could be associated with ORAI1 inhibition or over-activation in VSMC. The rapidly increasing knowledge of the implications of ORAI1 signalling in vascular remodelling holds promise to generate novel therapeutic tools for atherosclerosis and to prevent NIH following endovascular intervention.

## Author Contributions

HS, KN, and MB wrote the manuscript. CC and RF reviewed and edited the manuscript. DB provided intellectual input. All authors contributed to the article and approved the submitted version.

## Conflict of Interest

The authors declare that the research was conducted in the absence of any commercial or financial relationships that could be construed as a potential conflict of interest.
